# Microbiota activates IMD pathway and limits Sindbis infection in *Aedes aegypti*

**DOI:** 10.1186/s13071-017-2040-9

**Published:** 2017-02-23

**Authors:** Ana Beatriz Ferreira Barletta, Maria Clara L. Nascimento-Silva, Octávio A. C. Talyuli, José Henrique M. Oliveira, Luiza Oliveira Ramos Pereira, Pedro L. Oliveira, Marcos Henrique F. Sorgine

**Affiliations:** 1 Instituto de Bioquímica Médica Leopoldo de Meis, Universidade Federal do Rio de Janeiro, Rio de Janeiro, RJ Brazil; 2 Departamento de Microbiologia, Imunologia e Parasitologia, Universidade Federal de Santa Catarina, Florianópolis, SC Brazil; 3 Laboratório de Pesquisas em Leishmaniose, IOC, FIOCRUZ, Rio de Janeiro, RJ Brazil

**Keywords:** *Aedes aegypti*, Sindbis virus, Immune response, IMD, Microbiota

## Abstract

**Background:**

*Aedes aegypti* is the main vector of important arboviruses such as dengue, Zika and chikungunya. During infections mosquitoes can activate the immune pathways Toll, IMD and JAK/STAT to limit pathogen replication.

**Results:**

Here, we evaluate the immune response profile of *Ae. aegypti* against Sindbis virus (SINV). We analyzed gene expression of components of Toll, IMD and JAK/STAT pathways and showed that a blood meal and virus infection upregulated aaREL2 in a microbiota-dependent fashion, since this induction was prevented by antibiotic. The presence of the microbiota activates IMD and impaired the replication of SINV in the midgut. Constitutive activation of the IMD pathway, by Caspar depletion, leads to a decrease in microbiota levels and an increase in SINV loads.

**Conclusion:**

Together, these results suggest that a blood meal is able to activate innate immune pathways, through a nutrient induced growth of microbiota, leading to upregulation of aaREL2 and IMD activation. Microbiota levels seemed to have a reciprocal interaction, where the proliferation of the microbiota activates IMD pathway that in turn controls bacterial levels, allowing SINV replication in *Ae. aegypti* mosquitoes. The activation of the IMD pathway seems to have an indirect effect in SINV levels that is induced by the microbiota.

**Electronic supplementary material:**

The online version of this article (doi:10.1186/s13071-017-2040-9) contains supplementary material, which is available to authorized users.

## Background

The mosquito *Aedes aegypti* is the vector of important arthropod-borne viruses (arboviruses), such as dengue, chikungunya and Zika virus. Dengue is endemic in at least 100 countries in Asia, the Pacific, the Americas, Africa and the Caribbean [1]. The recent emergence of chikungunya and Zika virus in South America raised a red flag concerning the control of diseases transmitted by mosquitoes. Sindbis virus (SINV) is an arbovirus within the genus *Alphavirus* (Family *Togaviridae*), the same family and genus of the chikungunya virus and could be a useful tool to study the interaction of alphaviruses with the invertebrate host. SINV natural transmission is frequent in Eurasia, Africa, Oceania and Australia, and are usually passed from birds to humans through mosquitoes [2]. Since there is no available vaccine or medicine to effectively treat sick individuals, anti-arboviral strategies rely heavily on vector control, mainly through the use of insecticides. However, this approach is losing efficacy because of the rapid development of resistance by this vector [3]. This scenario reinforces the need to understand the biology of vector/pathogen interaction aiming to develop vector control strategies.

The Toll, Immune Deficiency (IMD) and Janus Kinase (Jak) signal transducer and activator of transcription (STAT) are major insect immune pathways activated by bacteria, fungi and viruses [4]. In *Ae. aegypti,* the Toll pathway is involved in responses against multiple pathogens, such as dengue virus [5–7]. Engagement of mosquito Toll involves the participation of the adaptor protein aaMYD88 (AAEL007768), the NFkB-like transcription factor aaREL1 (AAEL007696), its negative regulator aaCactus (AAEL000709) and aaSerpin (AAEL007765), a serine protease inhibitor [6, 8]. The IMD pathway was shown to involve the participation of aaIMD (AAEL010083), the transcription factor aaREL2 (AAEL007624) and the antimicrobial peptide defensin A, aaDef, (AAEL003841), among others, and protects mosquitoes against gram positive and negative bacteria in *Aedes* sp. and against *Plasmodium* infections in *Anopheles gambiae* [9–13]. In *Drosophila melanogaster,* the Jak/STAT pathway is triggered by unpaired (UPD) peptide binding to DOME receptor and leads to translocation of STAT (AAEL009692) dimer into the nucleus activating expression of several genes, like thioester-containing protein 1 (TEP-1) (AAEL001794) [14, 15]. The Jak/STAT pathway has an antiviral role well described in *Ae. aegypti* mosquitoes in response to dengue infection [16, 17].

The gut microbiota also influences the immune responses against pathogens [18–20]. In *D. melanogaster*, the intestinal homeobox gene *Caudal* regulates gut microbiota by repressing Relish-dependent AMP expression. When *Caudal* is suppressed, the IMD pathway is over activated leading to disruption of commensal microbiota, gut epithelial cell apoptosis and host mortality [21]. In *Ae. aegypti*, microbiota depletion using antibiotic treatment leads to an increase in dengue virus infection [7], indicating that this tripartite relationship influences the efficiency of the mosquito immune system and impacts directly the outcome of the infection [22].

Here, we investigated the activation of the three main pathways of *Ae. aegypti* immune system through the expression profile of their transcription factors (aaREL1, aaREL2 and STAT), adaptor proteins (aaIMD, aaMYD88) and known effector molecules (aaDefensin, aaTEP and aaSerpin) after SINV infection. We also addressed the roles of gut bacteria in immune activation and in response to SINV infection. Our results reveal that the activation of *Ae. aegypti* IMD pathway against SINV infection is highly dependent on the microbiota present in the midgut. IMD constitutive activation leads to a decrease in the microbiota levels allowing SINV increase in the mosquito. We propose that the IMD pathway has an indirect effect on SINV levels by controlling microbiota.

## Methods

### Mosquitoes and artificial meals

*Aedes aegypti* Red-eye strain mosquitoes were reared at 28 °C, 70–80% humidity in a 12:12 light:dark photoperiod. Mosquitoes were fed with sucrose 5% *ad libitum*. For artificial meals, 4–8 days old females were starved of sucrose, but not water, for 18–24 h. Mosquitoes were allowed to feed until repletion (1 h) in an artificial feeder kept at 37 °C and then were anaesthetized by cold. Fully engorged females were separated on ice and put into a new cage. Females were maintained in the same conditions as before for different periods of time until RNA extraction. All experiments were repeated at least three times.

### SINV propagation and stocks

SINV strain AR339 was propagated in C6/36 cells: Cells seeded to 80% confluency in 75 cm^2^ flasks were infected with virus stock at a multiplicity of infection (MOI) of 3.5, and incubated for 6 days at 32 °C and 5% CO_2_. Infected cells were scraped into solution and lysed to release virus particles by repeated freezing and thawing in dry ice and a 37 °C water bath. For the control groups, we did the same procedure as the infected cells but with C6/36 culture not infected (mock condition).

### SINV infection and titration by plaque assay

SINV infections were adapted from Lanciotti protocols for dengue virus infection [23]. Briefly, 350 μl of red blood cells from rabbit previously washed with phosphate saline buffer with 150 μl of 10^9^–10^14^ pfu/ml virus stock or 150 μl of virus-free mock media and were offered in an artificial feeder as described above. Mosquitoes were maintained under the same conditions as before. After 7 days post-infection, individual mosquitoes were homogenized in DMEM with a pestle, serially diluted, and then inoculated onto BHK cells seeded to 80% confluency in 24-well plates (100 μl per well). Plates were rocked for 15 min at room temperature, and then incubated for 45 min at 37 °C and 5% CO_2_. Subsequently, 1 ml of DMEM containing 2% FBS and 0.8% methylcellulose (Sigma No. M0512. Viscosity 4,000 cP) was added to each well, and plates were incubated for 2 days at 37 °C and 5% CO_2_. Plates were fixed with a methanol/acetone mixture (1∶1 volume) for >1 h at 4 °C, and plaque-forming units were visualized by staining with 1% crystal violet solution for 10 min at room temperature.

### Antibiotic treatments and 16S expression

To reduce/eliminate gut bacteria, penicillin (100 U/ml) and streptomycin (0.1 mg/ml) were added to the 5% sucrose solution used to feed insects, which was changed daily for 4 days before and 4 days after blood meal. Prior to the dissection, the surface of the mosquitoes was sterilized first with bleach for 1 min followed by one wash with sterile phosphate saline buffer (137 mM NaCl, 2.7 mM KCl, 10 mM Na_2_HPO_4_, 1.8 mM KH_2_PO_4_). Next, mosquitoes were washed in 70% ethanol for another minute and then washed in sterile phosphate saline buffer. Then, mosquitoes were dissected and five midguts from each condition were homogenized and plated onto a LB-agar medium under aseptic conditions. Bacteria colonies were incubated at 37 °C for 24 h to control for efficacy of antibiotic. For 16S expression, RNA pools of 5 mosquitoes were extracted 4 days post SINV infection. The bacterial load was calculated in all conditions in comparison with RP-49 gene (Ribosomal protein 49) expression [24].

### Gene-silencing assays

Experiments were performed by injecting approximately 69 nl of a double-stranded RNA (dsRNA) solution (3 μg/μl) in water into the thorax of anesthetized 4 days old female mosquitos using a nano-injector (Nanoject II - nanoliter injector, Drummond Scientific Company). After 24 h post-injection, mosquitoes were infected with SINV by ingesting an infectious blood meal. Pools of 5 mosquitoes were homogenized 4 days after infection for quantification of the microbiota loads, using 16S expression by qPCR. Viral loads were measured 7 days post-infection in individual mosquitoes by qPCR. The oligonucleotides used for viral and bacterial quantification were listed in Table 1. DsRNA solutions for Caspar and LacZ-control were obtained by reverse transcription using the T7 mega script kit (Ambion). A pCRII-TOPO plasmid containing a cloned fragment of the LacZ gene (218-bp) was used to obtain the template for dsRNA control synthesis [25].

**Table 1:** List of primers used in this study

Accession number	Gene	Primer sequence (5’–3’)
AAEL007696	aaREL1 For	GACTCGTCGGAGCTGAAATC
	aaREL1 Rev	CGGTTTGTTCAGGTTGTTGA
AAEL007624	aaREL2 For	TCTGTCGGCAGATGAAGTGA
	aaREL2 Rev	GCACTGGAATGGAGAATCAAA
AAEL000709	Cactus For	TCTTGCGTTGAAGTGAGTGG
	Cactus Rev	GACCCTCTGAAAGGGAAAGG
AAEL003841	Defensin For	GATTCGGCGTTGGTGATAGT
	Defensin Rev	TTATTCAATTCCGGCAGACG
AAEL010083	IMD For	TCGTCAAACTCGGTTTTCCT
	IMD Rev	TGGCGGAGTTGAAGGTAAAG
AAEL007768	Myd88 For	CGATGCGTTCATTTTGTTTG
	Myd88 Rev	CACCGCTCAGAAATCAGCTT
AAT45939	RP49 For	GCTATGACAAGCTTGCCCCCA
	RP49 Rev	TCATCAGCACCTCCAGCT
AAEL005673	Serpin For	ACGTGATGGATTGGATGGAG
	Serpin Rev	GTGCCTGCACTTGTTTCTGA
AAEL009692	STAT For	CACACAAAAAGGACGAAGCA
	STAT Rev	TCCAGTTCCCCTAAAGCTCA
AAEL001794	TEP For	ATTTTTGACGGCTTTTGTGG
	TEP Rev	TGGATTACTTGCCCCACTTC
Sindbis virus quantification primer	SINV For	TGACTAACCGGGGTAGGT
	SINV Rev	TTGGCTTCGGTGGGCATC
AAEL003579	dsCaspar For	TAATACGACTCACTATAGGG* GGAAGCAGATCGAGCCAAGCAG
	dsCaspar Rev	TAATACGACTCACTATAGGG* GCATTGAGCCGCCTGGTGTC
16S (For bacteria quantification)	16S For	TCCTACGGGAGGCAGCAGT
	16S Rev	GGACTACCAGGGTATCTAATCCTGTT
M13 (LacZ fragment)	M13 For	GTAAAACGACGGCCAGT
	M13 Rev	CTCGAGTAATACGACTCACTATAGGGCAGGAAACAGCTATGAC

*T7 tail is showed in bold

### RNA extraction and cDNA synthesis

RNA was isolated from pools of five females (whole body samples) or tissues were dissected in phosphate buffer. RNA was extracted using TRIzol reagent (Invitrogen) according to the manufacturer’s instructions. Total RNA was quantified in a spectrophotometer and 1 μg was treated with 1 U of DNAse RNAse free (Invitrogen) for 30 min at 37 °C. Reactions were stopped by adding 1 μl of 20 mM EDTA and heating for 10 min at 65 °C. cDNA synthesis was performed from the DNAse treated RNA according to High Capacity cDNA Reverse Transcription Kit from Applied Biosystems.

### Primers and PCR

All primers were made using Primer 3 (http://bioinfo.ut.ee/primer3-0.4.0/) and Oligo analyzer software (https://www.idtdna.com/calc/analyzer) (Table 1). Primer quality was accessed in a previous work [5].

Before real time PCR reactions all primer pairs were tested by conventional PCR using a 40 cycles reaction (denaturation at 94 °C for 30 s; annealing at 60 °C for 30 s; Taq extension at 72 °C for 30 s) in a thermocycler and analyzed in 2% agarose gel. Quantitative PCR (qPCR) was performed in an ABIXX (Applied Biosystems) using power SYBR-GREEN PCR master MIX. The Comparative ΔΔCt Method was used to compare changes in gene expression levels [26, 27]. RP-49 gene (Ribosomal protein 49) was used as endogenous control [24].

### Statistical analysis

Statistical analyses were made using ANOVA and Student’s *t*-test of ΔΔCt values. The parameters of all the statistical analysis are combined in Additional file 1 (supplementary material).

## Results

### The IMD pathway is transcriptionally activated after a blood meal

**Fig. 1:**
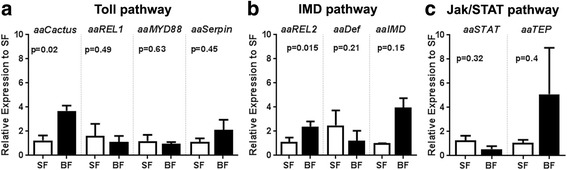
Whole body gene expression of Toll and IMD pathway genes are induced upon blood-feeding. Gene expression of the three main mosquito immune pathways was evaluated by qPCR using whole body samples 24 h post blood-feeding. **a** Toll pathway components. **b** IMD pathway components. **c** Jak/STAT pathway components. The blood-fed samples were compared with sugar fed samples. Relative expression was calculated using the ΔΔCt method using sugar fed expression as reference. RP49 gene was used as a reference gene. We performed Student’s *t*-test for statistical analysis between sugar and blood-fed conditions for each gene analyzed

**Fig. 2:**
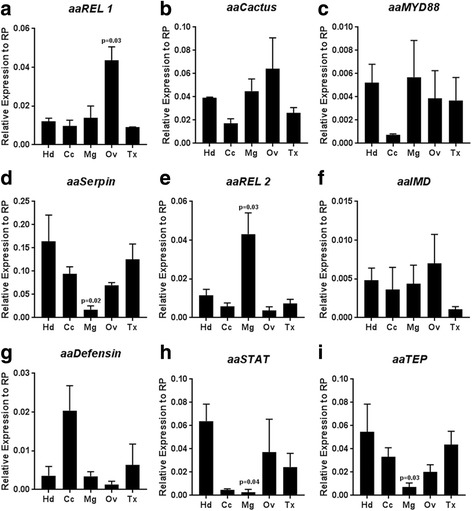
Transcription factor aaREL2 is upregulated in response to blood-feeding in the midgut of mosquitoes. Gene expression of the three main mosquito immune pathways was evaluated by qPCR in multiple tissues 24 h post blood-feeding. **a** aaREL1. **b** aaCactus. **c** aaMYD88. **d** aaSerpin. **e** aaREL2. **f** aaIMD. **g** aaDefensin. **h** aaSTAT. **i** TEP. Relative expression was calculated using the ddCt method using the lower tissue expression as reference. RP49 gene was used as a reference gene. We performed One-way ANOVA Tukey multiple comparison test for statistical analyses. *Abbreviations*: Hd, Head; Cc, carcass; Mg, Midgut; Ov, ovary; Tx, thorax

To investigate the possible role of blood-feeding on innate immune activation, we analyzed mRNA expression of key genes from the three major immune pathways after blood intake in whole body samples. At 24 h after blood-feeding, aaCactus, the inhibitor of the TOLL pathway was the only upregulated gene from this pathway (Fig. 1a). The transcription factor, aaREL1, the protein adaptor, aaMYD88, and one of the effector genes of this pathway, aaSerpin, did not show any altered regulation 24 h after a blood meal (Fig. 1a). The IMD pathway exhibited the strongest regulation among the three pathways, where the transcription factor aaREL2 and the protein adaptor aaIMD were induced 24 h after blood ingestion (Fig. 1b). The transcription factor aaSTAT was slightly downregulated while the effector gene of this pathway, aaTEP, showed a trend of induction (Fig. 1c). Although expression profile of the whole body suggested an activation of IMD pathway following a blood meal, we further investigated the expression profile of isolated mosquito tissues to have a clear picture of the immunological changes following blood ingestion. Hence, the mRNA expression profile of five different tissues (head, carcass, midgut, ovaries and thorax) was analyzed 24 h after blood meal. The transcription factor aaREL1, showed a significant induction in the ovaries, in comparison to the other tissues tested (Fig. 2a). Expression of aaMYD88 and aaCactus was not altered by blood ingestion in any tissue (Fig. 2b, c), while the expression of aaSerpin was significantly reduced in the midgut upon blood-feeding (Fig. 2d). Confirming the results obtained using whole body, the transcription factor aaREL2 was strongly induced by blood ingestion in the midgut (Fig. 2e). The aaIMD was not regulated in any of the tissues and aaDefensin, as expected, was highly induced in the carcass (Fig. 2f, g). Genes from the Jak-STAT pathway aaSTAT and aaTEP presented similar expression profile with low expression in the midgut and a tendency of increase in the head after blood ingestion (Fig. 2 h, i).

### aaREL2 expression is induced in the midgut in response to SINV infection

**Fig. 3:**
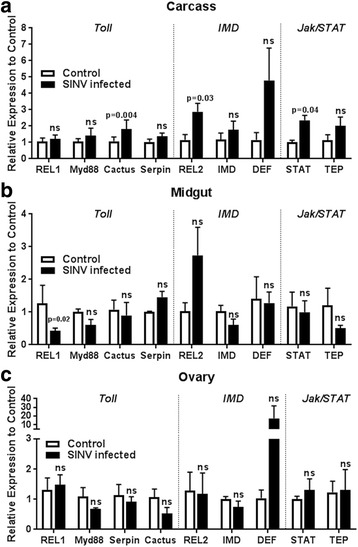
SINV infection induces expression of molecules from Toll, IMD and Jak/STAT pathways. Gene expression of the Toll, IMD and Jak/STAT pathways were evaluated by qPCR in three different tissues: carcass, midgut and ovaries. Tissues were dissected for RNA extraction 4 days post-infection. **a** Carcass. **b** Midgut. **c** Ovary. Relative expression was calculated using the ΔΔCt method comparing each infected tissue with the respective non-infected one, represented as 1 (blank bars). We performed Student’s *t*-test for statistical analysis between tissues infected and non-infected. RP49 was used as a reference gene

Next, we evaluated the impact of SINV infection in the transcription profile of the three main pathways, using carcass, midgut and ovaries 4 days after an infectious meal. For Toll pathway genes, aaREL1 expression showed a significant decrease in the midgut and aaCactus was induced in the carcass (Fig. 3a, b). No significant changes were observed for aaMyD88 and aaSerpin. (Fig. 3a, b). In the IMD pathway, we observed a positive regulation of aaREL2 in the infected carcass and midgut (Fig. 3a, b). In Jak-STAT pathway, SINV infection promoted an upregulation of both genes tested, aaSTAT and aaTEP, in the carcass (Fig. 3a). Similar to aaREL1, the transcription factor aaSTAT was not induced in the midgut in response to infection (Fig. 3b). In the ovaries, infection did not change the expression profile, except for an induction in aaDenfesin, 4 days after infection (Fig. 3c).

### The IMD pathway is transcriptionally activated by the gut microbiota after a blood meal

**Fig. 4:**
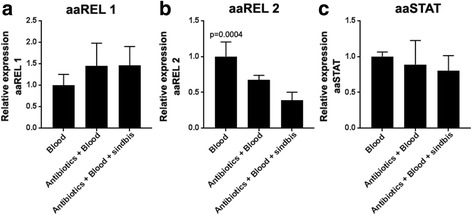
Microbiota depletion impacts aaREL2 expression in response to SINV infection. Relative gene expression of the transcription factors of Toll, IMD and Jak/STAT pathways was evaluated in response to microbiota depletion and SINV infection. Mosquitoes were either fed with blood, pretreated for 3 days with a sugar solution containing antibiotic (penicillin and streptomycin) and after that fed with blood or infected blood containing SINV particles. **a** Relative gene expression of aaREL1 4 days post-infection. **b** Relative gene expression of aaREL2 4 days post-infection. **c** Relative gene expression of aaSTAT 4 days post-infection. Relative expression was calculated using the ΔΔCt method and setting the blood condition as reference for comparison. RP49 was used as a reference gene. We performed One-way ANOVA Tukey multiple comparison test for statistical analyses

In order to evaluate how the gut microbiota contributes to the immune activation observed after blood-feeding and SINV infection, which accompanies the blood-feeding, mosquitoes were treated with antibiotic for 3 days before receiving a blood meal with or without SINV. Then, we analyzed gene expression levels of the transcription factors from the three major immune pathways 4 days post-infection. aaREL1 and aaSTAT mRNAs were not modified by antibiotic treatment, even during a SINV infection, suggesting their expression is not dependent on the microbiota (Fig. 4a, c). On the other hand, the reduction of gut bacterial levels, promotes a decrease in aaREL2 expression after both blood ingestion and SINV infection. This confirms that aaREL2 expression induction observed in Fig. 3e is dependent on the microbiota proliferation (Fig. 4b).

### The proliferation of the microbiota is crucial to limit SINV infection after blood ingestion

**Fig. 5:**
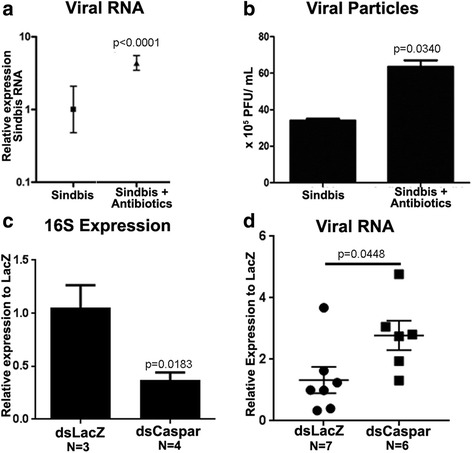
Microbiota depletion, by antibiotic treatment or IMD activation, leads to SINV particles increase in *Ae. aegypti.* After 4 days post-infection the amount of viral RNA and mature particles were measured using mosquito whole body samples. Mosquitoes were maintained in normal sugar or pretreated with antibiotic solution before feeding an infectious blood meal. **a** Viral RNA amounts relative to the reference gene (RP49). Mosquitoes maintained in normal sugar and then infected with SINV were set as 1. **b** Plaque assay of whole body samples from mosquitoes infected with SINV 7 days after infection. Mosquitoes injected with dsLacZ-control and dsCaspar were infected with SINV and the levels of bacteria and virus were measured by qPCR. **c** Four days post-infection the 16S expression was measured in pools of five mosquitoes. **d** After 7 days post-infection, SINV RNA was measured in whole body of individual mosquitoes. We calculated the relative expression of both bacteria and virus using the LacZ-control condition as a reference. Statistical analysis were conducted using Student’s *t*-test (unpaired)

Considering that antibiotic-treated mosquitoes presented lower expression levels of aaREL2, we analyzed SINV infection levels under these conditions. When compared to control mosquitoes, the antibiotic treated group presented about 6 times more viral RNA, (Fig. 5a), and 3 times more viral infective particles (Fig. 5b), showing that the microbiota growth that follows a blood meal [28] is responsible for an activation of the IMD pathway impacting SINV loads in the mosquito.

### Activation of the IMD pathway by Caspar silencing increases SINV load and decreases microbiota levels

Next, we evaluated the effect of IMD activation in the amount of SINV and the microbiota levels in infected mosquitoes. To activate IMD constitutively we depleted the negative regulator of this pathway, Caspar, using RNAi gene silencing followed by SINV infection. Four-days-old mosquitoes were injected with dsCaspar and dsLacZ-control and 24 h post injection mosquitoes received an infectious blood meal. After 4 days post-infection, pools of 5 mosquitoes were collected for bacterial load measurement by qPCR, using 16S expression. Mosquitoes injected with dsCaspar and constitutively active for the IMD pathway presented lower levels of 16S, half of the bacterial amount, in comparison with LacZ injected controls (Fig. 5c). We also measured SINV viral load in mosquitoes injected with dsCaspar and dsLacZ control 7 days post-infection. The amount of SINV was measured in individual mosquitoes using specific primers for SINV by qPCR. We observed a 2-fold viral load increase in Caspar silenced mosquitoes in comparison to LacZ-control mosquitos (Fig. 5d).

## Discussion

**Fig. 6:**
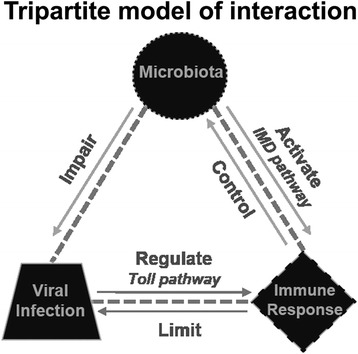
Tripartite model of interaction between the microbiota, virus and the insect immune system

The endogenous bacterial flora of insect midgut has a physiological role in nutrition, development, digestion, pathogen resistance and reproduction [29]. Recently, the mosquito microbiota has been implicated as an active component in the regulation of the immune system [30]. A reciprocal tripartite interaction between the microbiota, the immune system and dengue virus establishes that the microbiota elicits a basal immune activity that acts against virus infection after the blood intake [22]. Our results confirm this tripartite interaction and show that this is not a special feature of dengue virus but might be a general trend implicated in other mosquito-arboviruses systems (Fig. 6). Here, we demonstrated the role of the midgut microbiota in the induction of the IMD pathway after a blood meal and its anti-SINV effect. We addressed this by measuring the levels of gene expression of different components of Toll, IMD and Jak-STAT, and found that the blood meal induced the expression of aaREL2 in the midgut and whole body.

In mosquitoes, the Toll pathway has been identified as an important part of immune response against dengue virus. Microarray analysis revealed upregulation of Toll pathway related components, such as CLIP genes, and antimicrobial peptides (AMP), such as cecropins, defensin and gambicin [7]. The constitutive activation of the Toll pathway, by Cactus silencing, decreases dengue infection in *Ae. aegypti* midguts. In Aag-2 cells, a cellular model for immunity studies in *Ae. aegypti* [5], dengue virus infection downregulates the expression of Toll and Jak-STAT pathway components, but has no impact in expression of IMD regulated transcripts [31] (Fig. 6). This compiling evidence points to a direct relation between the Toll pathway and dengue infection, where the direct activation of this pathway is enough to limit infection in the midgut [7]. On the other hand, there must be a regulation of the Toll pathway components by the virus itself where infected cells downregulate Toll pathway related genes [31] (Fig. 6). Microarray analysis revealed that SINV infection also downregulates Toll pathway related genes 4 days post-infection suggesting that the regulation of this pathway by viral infection is similar between flaviviruses and alphaviruses [32]. So far, the effector molecules of Toll pathway involved in direct antiviral dengue response are still unknown. SINV transcript levels in antibiotic-treated mosquitoes 4 days post-infection were significantly higher than in the non-treated group. We suggest that the decrease observed in aaREL2 transcript after elimination of microbiota might account for this increased viral susceptibility observed in antibiotic-treated mosquitoes (Fig. 4b). In this way, we suggest that the expansion of the gut microbiota that follows a blood meal [28] could lead to the activation of the IMD pathway controlling SINV growth. We could not discard the role of the microbiota itself in the impact of SINV infection outcome.

The IMD constitutive activation by depletion of Caspar, the negative regulator of this pathway, leads to a decrease in the microbiota levels and allows SINV replication in the mosquito (Fig. 5c, d). Previous work indicates that depletion of Cactus and consequent constitutive expression of the Toll pathway leads to a decrease in microbial loads and dengue viral particles, pointing to a direct effect of the Toll pathway activation in anti-dengue response [7]. Here, we suggest that the constitutive activation of IMD pathway by Caspar silencing has an indirect impact SINV loads in the mosquito, by controlling microbiota levels that antagonize SINV infection.

In *Anopheles gambiae*, blood-feeding itself can trigger an anticipatory immune response against *Plasmodium berghei* infection [33]. However, different from our results, this anticipatory immune response is not due to microbiota proliferation, but has a hormonal component probably triggered by ecdysone release that follows blood ingestion [33].

Here we show evidence of the reciprocal tripartite interaction proposed by Ramirez et al. [22], where the proliferation of the microbiota could both activate the immune response, mainly the IMD pathway, and through this activation limit SINV infection. The proliferation of the bacteria itself could antagonize virus infection. We propose that the activation of the IMD pathway by the microbiota prevents its overgrowth and allows SINV replication (Fig. 6). This two-way regulation between the microbiota and the IMD pathway affects midgut conditions and could turn the mosquito permissive to infection.

Microarray analysis in *Ae. aegypti* midgut 4 days post SINV infection revealed a decrease in expression of ubiquitin ligase genes, suggesting not only the inhibition of Toll pathway activation, but also the activation of the IMD pathway [32]. These findings suggest a role of IMD pathway during SINV infection. Sanders et al. [32] is a remarkable piece of work to understand the global scenario during viral infection and points a possible role of the IMD pathway. Nonetheless the role of the microbiota, as well as its interaction in the activation of the immune system during an alphavirus infection has never been addressed. Our results add more evidence to Sanders et al. previous work acknowledging also the role of the IMD pathway in its tripartite interaction with the microbiota and SINV. These data could be important to understand the relationship between *Aedes* sp. and alphaviruses that recently became medically important, such as chikungunya. Nevertheless, additional studies will be required to demonstrate if this activation is specific for the control of SINV or if it can also be involved in the control of other virus, such as chikungunya and Zika.

## Conclusions

Our results suggest that blood meal ingestion is able to activate the IMD pathway, through a nutrient induced growth of microbiota, leading to upregulation of aaREL2. The activation of the IMD pathway is likely to be controlling microbiota levels in the midgut and consequently, allowing SINV replication in the mosquito. These results point to a tripartite interaction between the microbiota, the IMD pathway and SINV, where the microbiota levels are controlled by the IMD pathway and antagonize SINV replication in the mosquito. These data open a new venue to studies involving other viruses, like chikungunya and Zika, and their interaction within the invertebrate host.

## Additional file

Additional file 1: Detailed results from statistical analyses. (DOCX 13 kb)

## Abbreviations

AMP: Antimicrobial peptides
IMD: Immune deficiency
JAK: Janus kinase
MOI: Multiplicity of infection
TEP: Thioester containing protein
