# Supplementary material for: Microbiota activates IMD pathway and limits Sindbis infection in Aedes aegypti
Source: Parasit Vectors. 2017 Feb 23;10:103. doi: 10.1186/s13071-017-2040-9 (PMC5324288; doi:10.1186/s13071-017-2040-9)
Supplement: Additional file 1: — Detailed results from statistical analyses. (DOCX 13 kb) [file 13071_2017_2040_MOESM1_ESM.docx]

**Figure 1a**

aaCactus: t-test; t=6.683; df=2; p=0.0217

aaREL 1: t-test; t=0.7806; df=3; p=0.4919

aaMYD88: t-test; t=0.5487; df=2; p=0.6383

aaSerpin: t-test; t=0.9196; df=2; p=0.4548

**Figure 1b**

aaREL 2: t-test; t=8.076, df=2; p=0.0150

aaIMD: t-test; t=1.781; df=2; p=0.2169

aaDefensin: t-test; t=3.899; df=1; p=0.1598

**Figure 1c**

aaSTAT: t-test; t=1.179; df=3; p=0.3224

aaTEP: t-test; t=1.041; df=2; p=0.4071

**Figure 2a:** ANOVA; F=5.357; p=0.03

**Figure 2b:** ANOVA; F=3.091; p=0.0817

**Figure 2c:** ANOVA; F=1.904; p=0.1940

**Figure 2d:** ANOVA; F= 7.415; p=0.02

**Figure 2e:** ANOVA; F= 7.703; p=0.03

**Figure 2f:** ANOVA; F= 0.6262; p=0.6372

**Figure 2g:** ANOVA; F=2.168; p=0.1896

**Figure 2h:** ANOVA; F=4.123; p=0.04

**Figure 2i:** ANOVA; F= 5; p=0.03

**Figure 3a (Carcass)**

aaREL 1: t-test; t=1.76; df=3; p=0.1766

aaMYD88: t-test; t=1.366; df=3; p=0.2654

aaCactus: t-test; t=7.765; df=3; p=0.004

aaSerpin: t-test; t=2.339; df=3; p=0.1013

aaREL 2: t-test; t=3.713; df=3; p=0.0340

aaIMD: t-test; t=2.251; df=3; p=0.1099

aaDefensin: t-test; t=2.799; df=3; p=0.0679

aaSTAT: t-test; t= 4.643; df=2; p=0.0434

aaTEP: t-test; t=4.213, df=2; p= 0.0520

**Figure 3b (midgut)**

aaREL 1: t-test; t=4.1; df=3; p=0.0262

aaMYD88: t-test; t=2.605; df=3; p=0.08

aaCactus: t-test; t=1.359; df=2; p=0.3070

aaSerpin: t-test; t=2.407; df=2; p=0.1378

aaREL 2: t-test; t=2.569; df=2; p=0.1240

aaIMD: t-test; t= 2.9885; df=2; p=0.0963

aaDefensin: t-test; t= 1.45; df=2; p= 0.2841

aaSTAT: t-test; t=0.3867; df= 2; p= 0.7363

aaTEP: t-test; t=2.375; df=2; p=0.1408

**Figure 3c (ovary)**

aaREL 1: t-test; t=1.13; df=3; p=0.3407

aaMYD88: t-test; t=1.517; df=2; p=0.2684

aaCactus: t-test; t=2.92; df=2; p=0.1000

aaSerpin: t-test; t=0.4736; df=3; p=0.6681

aaREL 2: t-test; t=0.9294; df=2; p=0.4508

aaIMD: t-test; t= 1.104; df=2; p=0.3847

aaDefensin: t-test; t= 2.348; df=1; p= 0.2563

aaSTAT: t-test; t=0.4301; df= 2; p= 0.7091

aaTEP: t-test; t=0.4414; df=3; p=0.6888

**Figure 4a:** ANOVA; F=0.123; p=0.8849

**Figure 4b:** ANOVA; F=21.84; p=0.0004

**Figure 4c:** ANOVA; F=2.397; p=0.1134

**Figure 5a:** t-test; F=73006; df=6; t=3.031; p<0.0001

**Figure 5b:** t-test; t=3.713; df=3; p=0.0340

**Figure 5c:** t-test; t=3.449; df=5; p=0.0183

**Figure 5d:** t-test; t=2.264; df=11; p=0.0448
